# Regulation and functions of RhoU and RhoV

**DOI:** 10.1080/21541248.2017.1362495

**Published:** 2017-11-30

**Authors:** Richard G. Hodge, Anne J. Ridley

**Affiliations:** Randall Division of Cell and Molecular Biophysics, King's College London, New Hunt's House, Guy's Campus, London, UK

**Keywords:** Rho GTPases, RhoU, RhoV, post-translational modifications, signal transduction, cell adhesion, cell migration

## Abstract

Rho GTPases play central roles in a wide variety of cellular processes, including cytoskeletal dynamics, cell adhesion and cell polarity. RhoU and RhoV are Rho GTPases that have some atypical properties compared with classical Rho family members, such as the presence of N- and C-terminal extension regions, unusual GDP/GTP cycling and post-translational modification by palmitoylation but not prenylation. Their activity and localization is regulated by the N-terminal and C-terminal regions, and so far no GEFs or GAPs have been identified for them. Similar to Rac and Cdc42, they interact with PAK serine/threonine kinases, and in the case of PAK4, this interaction leads to RhoU protein stabilization. In cells, RhoU and RhoV alter cell shape and cell adhesion, which probably underlies some of the phenotypes reported for these proteins *in vivo*, for example in heart development and epithelial morphogenesis. However, the molecular basis for these functions of RhoU and RhoV remains to be characterized.

## Introduction

**Figure 1.**
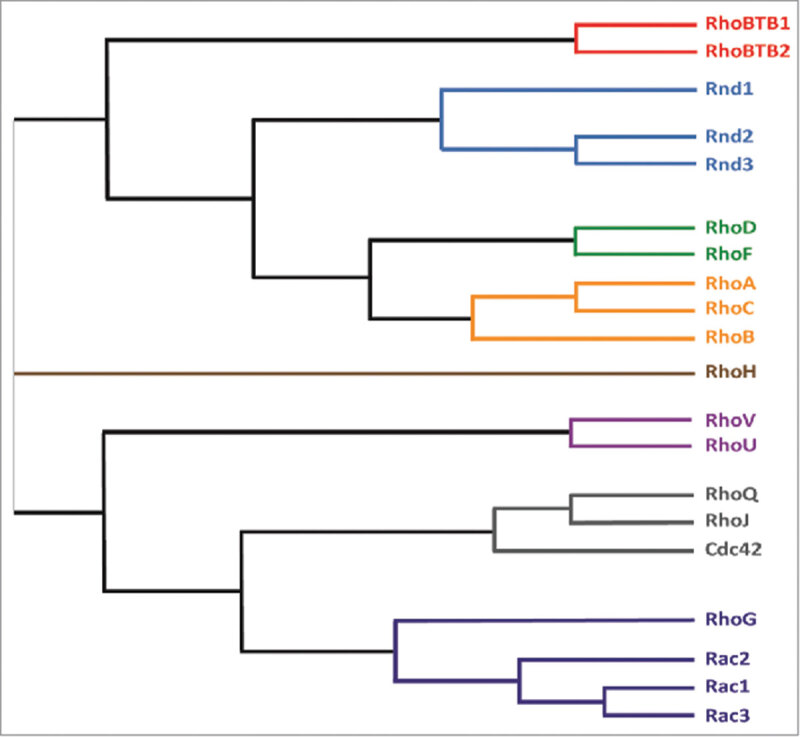
The Rho GTPase family. The 20 Rho GTPases are grouped into 8 distinct subfamilies based on sequence similarity. The phylogenetic tree was generated by Clustal Omega software (EMBL-EBI), using the amino acid sequences of the 20 Rho GTPases. The sequence of Rac1a and Cdc42 isoform 1 splice variants was used.

Rho GTPases are a distinct family within the Ras superfamily of small GTPases, based on their structure and function (Figure 1). Members of the family are present in all eukaryotic organisms sequenced to date. Rho GTPases are key regulators of the actin cytoskeleton, and have been shown to contribute to processes as diverse as cell migration, cell adhesion, cell polarity, cell division, transcriptional regulation and cell cycle progression.^1^ In humans there are 20 Rho GTPase genes, some of which have different splice variants.^2^

Most Rho GTPases cycle between an active GTP-bound conformation and an inactive GDP-bound conformation. This cycling is regulated by guanine-nucleotide exchange factors (GEFs), which stimulate release of GDP, allowing GTP to bind, and GTPase-activating proteins (GAPs), which catalyze GTP hydrolysis, hence inactivating the Rho GTPase. When bound to GTP, they interact with a range of downstream target proteins to induce cellular responses. The majority of Rho GTPases are post-translationally modified at their C-termini by addition of farnesyl or geranylgeranyl isoprenoid lipids, which mediate their interaction with cellular membranes. Some Rho GTPases also bind to RhoGDIs, which recognize geranylgeranylated proteins, extracting them from membranes and keeping them in an inactive complex in the cytosol.

RhoU and RhoV form a distinct subfamily of Rho GTPases based on their sequence homology (Figure 1). *RhoU* and *RhoV* genes emerged after *Rho, Rac* and *Cdc42* in evolution: whereas *Rho, Rac* and/or *Cdc42* genes are present in all eukaryotes, *RhoU* and *RhoV* are not found in plants or fungi or all animals, but a *RhoU/RhoV* ortholog is present in multiple Coelmate species, including insects and worms.^2^ The RhoU and RhoV proteins share 55.4% amino acid identity with each other.

*RhoU* (also known as *Wrch-1*: Wnt responsive Cdc42 homolog 1) was first cloned as a Wnt-inducible gene that induced the transformation of mouse mammary epithelial cells.^3^ RhoV (also known as Chp: Cdc42Hs homolog protein) was first discovered as a p21-activated kinase (PAK)2-interacting protein in a yeast 2-hybrid screen.^4^ *RhoU* mRNA appears to be expressed in most mouse tissues, whereas *RhoV* has a more restricted expression profile.^2^ RhoU and RhoV are considered to be atypical GTPases because they have several characteristics that distinguish them from the better characterized Rho family members such as RhoA, Rac1 and Cdc42. This review addresses the biochemical regulation and functions of RhoU and RhoV.

## RhoU and RhoV regulation and signaling

### Biochemical properties

RhoU is considered to be an atypical GTPase because it has been reported to have a 10-fold higher intrinsic guanine nucleotide exchange rate *in vitro* compared with Cdc42, and is therefore presumed to be predominantly GTP-bound in cells.^5,6^ By contrast, the biochemical characterization of RhoV GDP/GTP exchange or GTP hydrolysis has not been reported and therefore it is not known whether it exchanges GDP for GTP at a similarly high rate. Due to its homology with RhoU, RhoV is speculated to be similarly predominantly GTP-bound.^7^

### Role of N-terminal regions in regulating RhoU and RhoV

**Figure 2.**
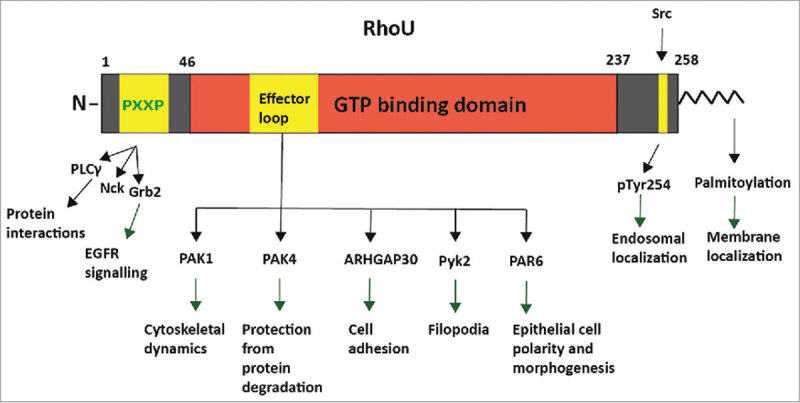
RhoU domain structure and protein interactions. RhoU has a core GTP-binding domain (red) and N- and C-terminal extension regions (gray). Regions that are known to interact with other proteins are indicated in yellow. The N-terminal region contains 3 proline-rich motifs (PXXP; yellow) which bind to SH3 domains in the indicated proteins. At the C-terminus is a CFV motif (amino acids 256–258) which is modified on the cysteine by palmitoylation and is required for membrane localization. The Src tyrosine kinase phosphorylates Tyr254, which alters RhoU localization. p21-activated kinases (PAKs) are well established RhoU effector proteins which bind to the effector loop (yellow) of RhoU and regulate cytoskeletal dynamics and cell migration. ARHGAP30, Pyk2 and PAR6 have also been described to bind to the effector loop by co-immunoprecipitation analysis and these interactions allow RhoU to regulate several cellular functions.

**Figure 3.**
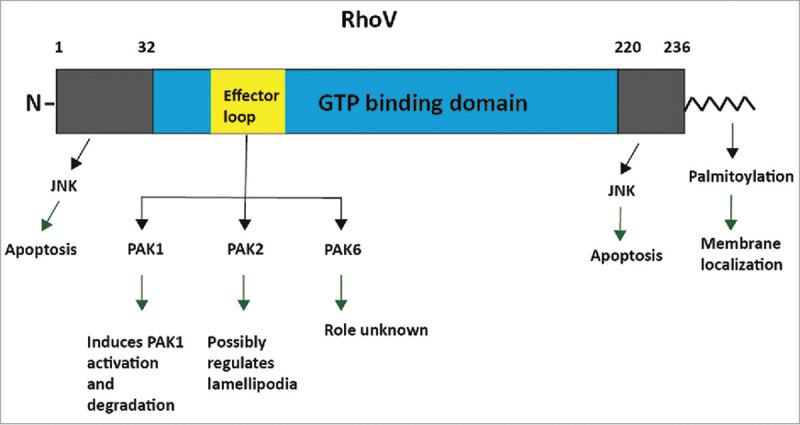
RhoV domain structure and protein interactions. RhoV has a core GTP-binding domain (blue) and N- and C-terminal extension regions (gray). The RhoV C-terminus has a CFV motif (amino acids 234–236) which is modified on the cysteine by palmitoylation. p21-activated kinases (PAKs) are well established effector proteins that bind to the effector loop (yellow), and mediate some of the functions of RhoV. RhoV has been reported to interact with PAK1, PAK2 and PAK6, although it has only been shown to activate PAK1. PAK2 interaction also requires the C-terminal extension. RhoV can regulate apoptosis in a JNK-dependent manner. The interaction with JNK has not been characterized biochemically, although deletion of the N-and C-terminal regions reduces JNK phosphorylation.

RhoU and RhoV have unique N-terminal and C-terminal regions either side of the core GTP-binding domain (Figures 2 and 3). Among the Rho GTPases, the 3 Rnd subfamily members also have N-terminal and C-terminal extensions.^8^

The N-terminal region has been proposed to act as a negative regulator of both proteins. N-terminal truncation of RhoU increases anchorage-independent growth of NIH3T3 fibroblasts and also enhances RhoU association with effector proteins.^5^ The N-terminal region of RhoU has been shown to inhibit its ability to activate PAK1, as indicated by PAK1 autophosphorylation.^5^ However, another study reported that RhoU required the N-terminal region to induce filopodia in porcine aortic endothelial (PAE) cells, and this region has proline-rich regions that bind to several proteins with SH3 domains (Figure 2). Hence, the role of the N-terminal region is controversial. Deletion of the N-terminal region does not alter the nucleotide exchange or GTP hydrolysis activity of RhoU in comparison with Cdc42.^5^

Similar to RhoU, deletion of the N-terminal region of RhoV resulted in growth transformation and an increase in anchorage-independent growth of NIH3T3 fibroblasts in soft agar.^9^ Furthermore, N-terminal deletion of RhoV resulted in the recruitment of a YFP-PAK-CRIB reporter to endosome structures. This indicates that the active pool of RhoV is found at endosomes, in contrast to a report on RhoU that suggested the endosomal pool was inactive.^10^ TNF-α activation was also shown to induce the activation of RhoV at the endosomal compartment, but not at the plasma membrane.^11^

### Role of C-terminal regions in sub-cellular localization

RhoU and RhoV are localized to both the plasma membrane and endomembrane compartments. Both RhoU and RhoV have been reported to be modified by C-terminal S-palmitoylation but unlike most other Rho GTPases are not prenylated^9,12^ (Figure 2 and 3). S-palmitoylation is a post-translational lipid modification in which a 16-carbon fatty acid palmitate group is covalently attached to cysteine residues on target proteins, and promotes their interaction with membranes.^12,13^ In contrast to protein prenylation, palmitoylation is a reversible enzymatic process. This allows RhoU and RhoV to associate transiently and dynamically with cellular membranes.^9,12^ Since RhoU and RhoV are not prenylated and therefore cannot bind RhoGDIs, the reversible nature of palmitoylation might serve to regulate their activity by altering their localization.^11,12^

In addition to S-palmitoylation, a stretch of polybasic residues and a tryptophan (W229) close to the C-terminus have been reported to facilitate the membrane localization and transforming potential of RhoV.^11^ Mutation of W229 resulted in localization to the cytoplasm, with an essentially identical localization pattern to C-terminally deleted RhoV.^11^ Two arginine residues near the C-terminus also affect RhoV localization: RhoV-R226Q and RhoV-R228Q have a slightly reduced plasma membrane localization but still associate with endomembranes.^11^ These C-terminal residues are also important for the recruitment of effector proteins to specific membrane sub-cellular locations. Deletion of the C-terminal region abolished the transforming activity of RhoV, indicating that the C-terminus is essential for its normal function.^9^

Src-mediated tyrosine phosphorylation of RhoU has been shown to regulate its sub-cellular localization. Phosphorylation of RhoU on a C-terminal tyrosine, Y254, results in a rapid relocalization of RhoU from the plasma membrane to the endosomal compartment.^10^ This was also associated with a decrease in GTP binding, and thus it was argued that the endosomal pool of RhoU is inactive. This was speculated to be due to altering its proximity to GEFs and GAPs, with a concomitant decrease in effector protein interactions.^10^ However, a separate study found that the RhoU-Y254F mutant was not phosphorylated by Src as expected but was still capable of localizing on endosomes.^14^ These 2 studies were performed in different cell types, and thus the contribution of tyrosine phosphorylation to RhoU localization might be cell-type dependent. Further studies are therefore required to determine how RhoU phosphorylation alters its function.

### Binding partners

**Figure 4.**
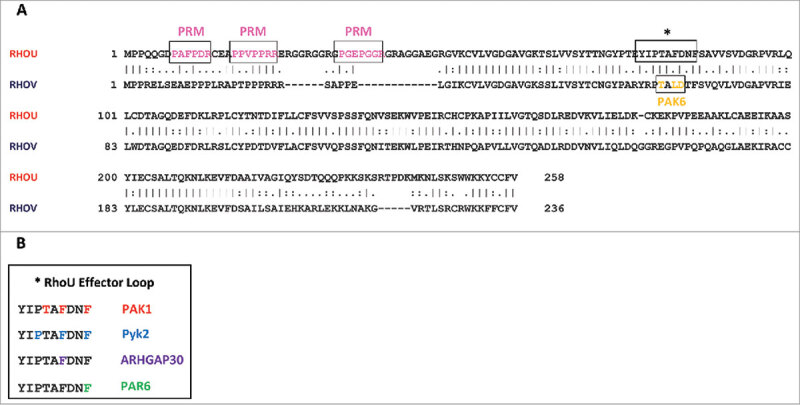
RhoU and RhoV effector protein interactions. (A) Human RhoU and RhoV protein sequences were aligned using the Clustal Omega pairwise sequence alignment tool (EMBL-EBI). Identical amino acids in the 2 sequences are indicated by a line, similar by 2 dots, and non-conserved by a dot. Proline-rich motifs in RhoU and specific residues that have been reported to interact with PAK6 (RhoV) are highlighted and boxed. The RhoU effector loop (asterisk-labeled box) is magnified in (B) and the residues responsible for interaction with the indicated effector proteins are highlighted. Mutation of T81, F83 and F86 in RhoU prevents PAK1 interaction but does not prevent interaction with PAK4. This indicates that distinct residues in the effector loops of RhoU and RhoV are responsible for the interaction with different PAK proteins. PRMs; proline-rich motifs; T, Thr; F, Phe; P, Pro.

RhoU and RhoV have been reported to interact with several proteins (Figure 2 and 3), of which the PAKs are the best-characterized. PAKs are serine/threonine-specific intracellular protein kinases that are involved in a variety of cellular responses, including changes in cell morphology, motility, survival and gene transcription, and are well known targets for Cdc42 and Rac1. RhoU activates PAK1^3,6^, and mutations in RhoU affect both its ability to bind to the PAK1 CRIB domain and to increase PAK1 autophosphorylation.^3,5^ RhoU also interacts with PAK4, although it appears to use different residues to interact with PAK4 compared with PAK1^15^ (Figure 4). Phospho-PAK4 (pS474) levels are unchanged upon RhoU overexpression, indicating that PAK4 is not a RhoU effector protein.^15^ Instead, PAK4 plays an unconventional kinase-independent role in RhoU regulation. PAK4 regulates RhoU expression levels by protecting RhoU from Rab40A-mediated proteasomal degradation.^15^ RhoU stabilization by PAK4 leads to stimulation of MDA-MB-231 breast cancer cell migration and promotes focal adhesion turnover.^15^

PAK2 and PAK6 have been shown to bind RhoV by co-immunoprecipitation analysis.^4,16^ Both PAK2 and PAK6 binding was increased by introduction of an activating mutation, RhoV-G40V, whereas binding was abolished with a RhoV-S45N dominant negative mutant.^4,16^ The C-terminal region of RhoV was necessary to mediate the interaction with PAK2, but the role of RhoV effector loop residues (switch I) was not investigated.^4^ On the other hand, RhoV was demonstrated to interact with the PAK6 CRIB motif using specific residues in its effector loop (T63, L65 and E66) (Figure 4), but the role of the N- and C-terminal extension regions was not tested. Interestingly, RhoV overexpression did not increase phospho-PAK6 (pS560) levels, suggesting that PAK6 is not a RhoV effector protein,^16^ similar to the observations with RhoU and PAK4.^15^ It would therefore be interesting to test whether PAK6 regulates RhoV stability.

As mentioned previously, the N-terminal region of RhoU contains 3 proline-rich motifs (PRMs). By contrast, RhoV does not contain PRMs in its N-terminal extension. The sequences in RhoU are PAFPDR (residues 8–13), PPVPPRR (20–26) and PGEPGGR (36–42) (Figure 4). The N-terminal region of RhoU binds to various SH3-domain containing adaptor proteins, including Grb2,^5,14^ Nck2^5,6^ and phospholipase Cγ.^5^ Peptide analysis has revealed that the central PxxP motif is responsible for mediating the interaction with Grb2 and Nck2.^17^ The interactions with Grb2, Nck2 and phospholipase Cγ are direct^5^ and are mediated by the PRMs located in the N-terminal region.^6,14^ Grb2 has been shown to couple RhoU to epidermal growth factor (EGF) receptor signaling, and RhoU co-localizes with the EGF receptor on endosomes after EGF stimulation.^14^ This allows RhoU to link to receptor tyrosine kinases and participate in downstream signaling cascades. It has been hypothesized that the binding of the adaptor proteins relieves the inhibitory roles of the N-terminal regions and promotes RhoU effector activation.^5^ Nck2 has also been shown to interact with PAK1, and thus it is possible that Nck2 serves to mediate the interaction between RhoU and PAK1 in cells.^6^

RhoU also associates with the non-receptor tyrosine kinase Pyk2, which requires the N-terminal region of RhoU.^18^ However, Pyk2 does not contain an SH3 domain, thus the interaction is unlikely to involve the PRMs, and it is unclear how Pyk2 interacts with RhoU. The FERM domain of Pyk2 was suggested to mediate the RhoU interaction, as well as specific residues in the RhoU effector loop (P80, F83 and F86) (Figure 4). Furthermore, Pyk2 was required for RhoU to induce the formation of filopodia. Src-mediated phosphorylation promoted the formation of the RhoU-Pyk2 complex and the subsequent activation of Pyk2,^18^ although whether this involves the RhoU C-terminal Src phosphorylation site Y254 (see above) is not known. Of note, Pyk2 might also phosphorylate RhoU, although the function of this phosphorylation event is unclear.

RhoU is speculated to be insensitive to GEFs due to its rapid guanine nucleotide exchange rate, but it could be regulated by GAPs. Indeed, RhoU co-immunoprecipitates with ARHGAP30 and CdGAP.^19^ Similar to RhoU overexpression, ARHGAP30 stimulated the formation of filopodia and stress fiber disassembly,^19^ suggesting that ARHGAP30 is downstream of RhoU signaling and that RhoU might stimulate ARHGAP30 activity.

## Functions of RhoU

### Cellular studies

RhoU overexpression has a striking effect on cell morphology and actin dynamics in a variety of cell models, although the effects appear to be cell-type dependent. RhoU is involved in promoting focal adhesion turnover, decreasing stress fibers , inducing filopodia and/or regulating cell adhesion in several cell types,^15,18,20^ but the mechanism underlying these RhoU-induced responses is not clear. In an osteoclast model, RhoU localized to podosomes, but not focal adhesion complexes, and this was dependent on the C-terminal region. RhoU was also demonstrated to increase the adhesion and migration of osteoclast precursor cells.^21^ In NIH3T3 fibroblasts, activated RhoU resulted in a significant loss of focal adhesions.^22^ In HeLa cells, RhoU also localized to focal adhesions and promoted their disassembly during cell migration.^20^ Conversely, siRNA-mediated knockdown of RhoU resulted in the reduced adhesion of T-ALL cell lines to fibronectin and to endothelial cells.^23^ This indicates that the role of RhoU in cell-substratum or cell-cell adhesion is cell-type dependent. However, another interpretation is that RhoU only reduces the assembly of large focal adhesion complexes, and since T-cells do not have focal adhesions it could have a different function in regulating adhesion in these cells.

RhoU has recently been reported to increase paxillin phosphorylation in MDA-MB-231 breast cancer cells.^15^ Paxillin is a scaffolding component in focal adhesion complexes and serves as a platform to integrate adhesion signaling.^24^ RhoU overexpression increased phospho-paxillin (pS272) levels in a PAK4-depleted background, and RhoU associated with paxillin.^15^ RhoU might provide a scaffold to recruit an additional kinase to induce paxillin S272 phosphorylation, possibly via JNK activation.^3,20^

RhoU has been implicated in regulating apicobasal epithelial cell polarity due to its ability to bind the polarity protein PAR6 via its effector loop (Figure 2).^25^ RhoU localized to the apical and basolateral membranes in MDCK epithelial cells and an activating RhoU-Q107L mutant was described to inhibit tight junction formation. Furthermore, a mutation in the RhoU effector loop prevented binding to PAR6 and was unable to delay tight junction formation, whereas the N-terminal extension was not required for this response. Cdc42 is well known to bind to PAR6 and stimulate apicobasal polarity^26^ but the effects and localization of RhoU in epithelial cells appear different to Cdc42, and thus direct comparison of the roles of the 2 Rho GTPases in epithelial polarity would be useful.

### *In vivo* studies

There is mounting evidence that RhoU regulates the polarity and architecture of cells during developmental processes.^27-29^ It contributes to the formation of cell-cell junctions between zebrafish cardiomyocytes through a pathway involving the GEF Arhgef7b and PAK1, thereby affecting cardiac morphogenesis.^27^ It would be interesting to determine whether PAR6 is involved in RhoU signaling in zebrafish cardiomyocytes, similar to its role in MDCK epithelial cells.^25^ In *C. elegans*, the RhoU/RhoV ortholog CHW-1 (which is equally similar to RhoU and RhoV) contributes to establishing polarity in vulval precursor cells (VPCs) during vulval development.^28^ Although loss of *chw-1* alone did not alter vulval development, it modulated the effects of 2 different Wnt receptors on VPC polarity,^28^ which might reflect its role as a Wnt-inducible gene.^3^

*RhoU* also plays a role in regulating the development of the mouse foregut.^29,30^ *RhoU* is expressed during embryogenesis in the epithelium of the developing gut. Once the epithelium becomes multi-layered *RhoU* expression decreases. Using embryos from *RhoU*-depleted embryonic stem cells, it was found that RhoU is required for epithelial morphogenesis. *RhoU*-depleted cells had fewer apical microvilli and a reduction in sub-apical filamentous actin levels. This indicates that RhoU is important for epithelial architecture,^29^ consistent with results *in vitro* with MDCK cells.^25^

Apart from the regulation of cell adhesion and cell shape during development, RhoU plays a role in cell migration in developmental models. Over-expression of the *C. elegans* RhoU/RhoV ortholog CHW-1 disrupted migration of gonadal distal tip cells,^27^ a well-characterized migration model in *C. elegans*.^31^ In addition, *RhoU* is required for the migration of cranial neural crest cells in *Xenopus laevis* embryos.^32^ *RhoU* also regulates the ability of these cells to differentiate into craniofacial cartilages.^32^

## Functions of RhoV

### Cellular studies

RhoV overexpression induces filopodia and lamellipodia in several different cell types.^4,33^ RhoV overexpression also induced the formation of focal adhesion complexes and localized to these sites in an endothelial cell line.^33^ This contrasts to the disassembly of focal adhesions stimulated by RhoU.

RhoV has been reported to activate the JNK MAPK kinase cascade in several studies.^4,34^ RhoV appears to stimulate the apoptosis of PC12 cells via activation of JNK and the death receptor-mediated and mitochondrial apoptotic pathways.^34^ RhoV mRNA expression increases as macrophages differentiate into osteoclasts, coinciding with a high level of apoptosis,^35^ but whether RhoV is responsible for this apoptosis is not known.

RhoV has an unconventional effect on PAK1. RhoV, as well as Cdc42, has been shown to stimulate the ubiquitination and proteasome-mediated degradation of PAK1 in Jurkat T- cells.^36^ RhoV interaction with and activation of PAK1 appears to be required for its degradation, based on the effects of RhoV on a range of PAK1 mutants Furthermore, both N-terminal and C-terminal extension regions of RhoV were required to trigger PAK1 degradation, and the N-terminal region was required for PAK1 degradation but not its activation. This suggests a second unknown function for RhoV in PAK1 degradation.^36^

### *In vivo* studies

RhoV has been described to be involved in developmental processes, although its function appears to differ from RhoU. For example, RhoU and RhoV have different functions in neural crest cells in *Xenopus laevis*.^37^ RhoV expression is transiently induced during neural crest differentiation by the canonical Wnt pathway. RhoV depletion inhibited the expression of neural crest markers such as the transcription factors Sox9, Slug and Twist and blocked differentiation. Conversely, RhoV overexpression induced expansion of the neural crest domain.^38^ On the other hand, RhoU is induced later by the non-canonical Wnt pathway and contributes to neural crest migration, as described above.

RhoV plays a role in maintaining E-cadherin at adherens junctions during zebrafish gastrulation.^39^ During gastrulation, the zebrafish embryo undergoes a series of morphological changes to form the 3 distinct germ layers: endoderm, mesoderm and ectoderm. E-cadherin-mediated cell-cell adhesion is essential during this process. RhoV depletion induced a strong phenotype in zebrafish gastrulation, similar to that induced by E-cadherin depletion. It reduced E-cadherin levels at cell-cell junctions, as well as phospho-PAK localization at these regions. PAK interacts with the Rac/Cdc42 GEF β-PIX, and depletion of β-PIX induced a similar gastrulation phenotype to RhoV depletion, suggesting that RhoV acts via PAK and β-PIX to localize E-cadherin.^39^ So far RhoV has not been reported to affect E-cadherin localization in cultured cells *in vitro*, and thus this would be an interesting area for future research.

## Conclusions and future perspectives

Despite the sequence similarity between RhoU and RhoV, they have several different functions in mammalian cells *in vitro* and in model organisms. So far, however, little is known of the molecules that activate RhoU and RhoV during developmental processes or cellular responses *in vitro*. Further information on post-translational modifications and the roles of known or yet-to-be-identified interacting partners in regulating their activity is needed. For example, it is unclear whether they are downregulated by GAPs or stimulate GAPs to act on other Rho GTPases. There is little evidence yet that implicates these proteins in cancer progression. However, RhoU has been described to be upregulated in T-cell acute lymphoblastic leukemia (T-ALL)^23^ and RhoV is upregulated in non-small cell lung cancer.^40^ Their roles in regulating cell migration and cell adhesion *in vitro* and *in vivo* make them potential targets in cancer progression.
